# Nafion: A Flexible Template for Selective Structuring

**DOI:** 10.3390/polym16060744

**Published:** 2024-03-08

**Authors:** Nikolai F. Bunkin, Polina N. Bolotskova, Sergey V. Gudkov, Minh T. Khuong, Valeriy A. Kozlov, Svetlana L. Timchenko, Valeriy V. Voronov, Yulia V. Novakovskaya

**Affiliations:** 1 Department of Fundamental Sciences, Bauman Moscow State Technical University, 2nd Bauman Str. 5, Moscow 105005, Russia; bolotskova@inbox.ru (P.N.B.); khuongthuminh@gmail.com (M.T.K.); v.kozlov@hotmail.com (V.A.K.); svtimchenko@yandex.ru (S.L.T.); 2 Prokhorov Institute of General Physics, Russian Academy of Sciences, Vavilov Str. 38, Moscow 119991, Russia; s_makariy@rambler.ru (S.V.G.); voronov@lst.gpi.ru (V.V.V.); 3 Chemistry Department, Lomonosov Moscow State University, Leninskie Gory, 1/3, Moscow 119991, Russia; jvnovakovskaya@gmail.com

**Keywords:** polymer membrane, Nafion, X-ray diffraction, –SO_3_H groups, deuterium-depleted water, crystal growth, stabilizing polymeric template

## Abstract

The peculiarities of crystal growth on a Nafion polymeric substrate from supersaturated aqueous solutions of initial substances were studied. The solutions were prepared based on deionized natural water and deuterium-depleted water. As was found earlier, in natural water (deuterium content 157 ± 1 ppm) polymer fibers are capable of unwinding towards the bulk of the liquid, while in deuterium-depleted water (deuterium content ≤ 3 ppm) there is no such effect. Since the distance between the unwound fibers falls in a nanometer range (which is close to the size of the unit cell of the crystal lattice), and these fibers are directed normally to the polymeric substrate, the unwinding can affect crystal growth on the polymer substrate. As was obtained in experiments with X-ray diffractometry, the unwound polymer fibers predetermine syngony of crystals, for which the unit cell is either a rectangular parallelepiped (monoclinic system) or an oblique parallelepiped (triclinic system). A quantitative theoretical model that describes the local interaction of the polymer substrate with the crystalline complexes is presented. Within this model, the polymer substrate can be considered as a flexible matrix for growing crystals.

## 1. Introduction

The basic interest in the perfluorinated polymer membrane Nafion™ is due to the wide use of this polymer in fuel cells for hydrogen energy. Being composed of perfluorinated hydrocarbon chains with side branches of perfluorinated polyether with –SO_3_H terminal groups and a general formula [1]:
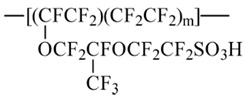
(1)
it involves fragments with drastically different response to water and aqueous solutions, namely, a highly hydrophobic Teflon™ skeleton, hydrophilic terminal groups, and somehow transient bridges in between, since polyether fragments are slightly hydrophilic. Such a combination of properties should be reflected in a characteristic response of the polymer material to water-based environments, being sensitive to minor variations in the state and/or composition of the system. Particularly, both normal and reverse micelles based on the material particles can be formed under certain conditions. This is not the sole interesting feature of the material that can be used in diverse fields. Nafion matrix is biocompatible and demonstrates excellent mechanical and chemical resistance.

In water, the polymer swells, and through channels with a diameter of 2 to 3 nm are formed in the bulk matrix (see [1] for more details). Terminal –SO_3_H groups dissociate to produce hydronium ions:

R–SO_3_H + H_2_O ⇔ R–SO_3_^−^ + H_3_O^+^ (2)

while the negatively charged residues provide the possibility of ion transport [2]. Here, R stands for the polymer chain. The nanoscale structure of the channels produces conditions for the separation of H^+^ and OH^−^ ions at the two membrane sides which is utilized in hydrogen cells [3]. The mechanisms of ion separation (see, e.g., review [4]) and the ion exchange processes inside the channels (e.g., [5]) are extensively studied. In both cases, conformations of the side perfluorinated vinyl ether-based chains should play an important role.

One of the techniques used for the investigation of the behavior of Nafion is luminescent spectroscopy (see, e.g., review [6]). Our experiments particularly showed that the character of the Nafion swelling in water depends on the deuterium content. For example, in [6,7] the swelling of a Nafion plate in water samples with a deuterium content of 3 ppm (so-called deuterium-depleted water, DDW) to 10^6^ ppm (heavy water) was investigated, and the distance between the polymer surface and the most distant area (with respect to the polymer surface) in the bulk water, where the luminescence is still nonzero, was measured. It turned out that in natural water (with a deuterium content of 157 ± 1 ppm, see [8]), the luminescence intensity is not leveled off up to distances of about 500 μm, while in DDW it dropped to zero at around 5 μm. As was found in [9], the surface bundles of polymer fibers of Nafion which is brought in contact with natural water are oriented basically normally to the Nafion–water interface, whereas in the environment of water vapor, these bundles are oriented tangentially to the interface. In [9], the Nafion surface was studied with an atomic force microscopy, which enabled the authors to estimate the mean distance between the fibers oriented normally to the polymer surface as several nanometers. These results indirectly confirm an idea about the partial unwinding of the polymer fibers in water.

The unwinding of the polymer fibers should result in some conformational changes in the polymer matrix. We assume that terminal SO_3_H groups are no longer tightly surrounded by more or less hydrophobic fiber segments, but rather brought into contact with water molecules. Thus, in addition to the aforementioned dissociation of SO_3_H groups, the internal configuration of the groups and their close neighborhoods (i.e., internuclear distances) should change, which means that the ground and excited electronic states should change as well. It is straightforward to assume that the absorption spectra of Nafion specimens soaked in deionized natural water and DDW should be different. Furthermore, if the electronic configuration of the polymer matrix depends on the deuterium content in water, this should manifest itself in the deposition processes at the Nafion interface, particularly in the growing of crystals at this interface as a substrate.

The goal of this work is to verify that upon swelling Nafion in water with different isotopic compositions, the absorption spectrum should depend on the deuterium content. The characteristic time of soaking at which this dependence is revealed should be found. To achieve this, we plan to conduct studies of the time dynamics of the absorption coefficient of Nafion during soaking in natural water and DDW. In addition, here we describe an experiment on growing crystals from supersaturated aqueous solutions of various substances on the Nafion substrate or on a smooth glass surface; aqueous solutions were prepared based on natural deionized water and DDW. Finally, we present the results of measuring the deposition rate from a supersaturated solution onto a Nafion substrate and a glass surface. A theoretical model that describes the local interactions of the polymer matrix with substances dissolved in water allowed us to substantiate the experimental results at a semi-quantitative level.

## 2. Methods and Materials

In the experiments, we used Nafion N117 plates with a thickness of 175 μm, purchased from Sigma Aldrich, St. Louis, MO, USA. Two kinds of water samples were used. One test liquid was deionized water (deuterium content 157 ± 1 ppm) with a resistivity of 18 MΩ·cm at 25 °C refined with a Milli-Q apparatus (Merck KGaA, Darmstadt, Germany). The other test liquid was deuterium-depleted water (DDW) with a deuterium content ≤ 3 ppm, purchased from Sigma Aldrich, St. Louis, MO, USA. The absorbance of Nafion in deionized natural water and DDW was measured with a PB 2201 spectrophotometer (SOLAR, Minsk, Belarus) with the following characteristics: spectral range is 190–1100 nm, spectral gap is 0.2–2.0 nm with a step of 0.1 nm, scanning speed is 5–10,000 nm/min, and wavelength setting accuracy is ±1.0 nm.

The following crystalline substances have been investigated: potassium chloride KCl, sodium acetate NaCH_3_COO, copper sulfate CuSO_4_, as well as sucrose C_12_H_22_O_11_. All the substances were purchased from Sigma Aldrich (St. Louis, MO, USA).

**Figure 1:**
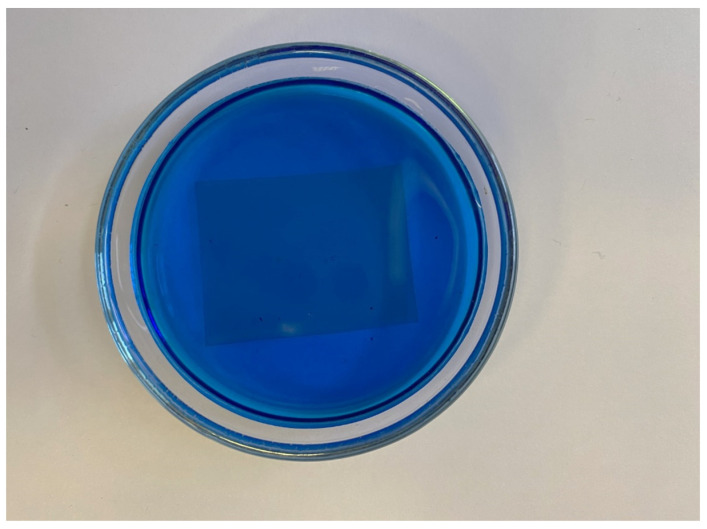
Nafion plate in an aqueous solution of CuSO_4_.

For the investigation of the peculiarities of the crystals which were grown on the Nafion substrate from the supersaturated aqueous solutions of these salts, the techniques of X-ray diffractometry (XRD) and refractometry were used. In these experiments, a Nafion N117 plate with an area of 3 × 2.5 cm^2^ was placed in a Petri dish filled with an aqueous solution of the tested salt with a volume of 25 mL and a concentration close to the saturation threshold at 20 °C. The solutions were prepared based on either natural deionized water or DDW. In the reference experiments, the same solutions were poured into the Petri dish in the absence of a Nafion plate. Uncovered Petri dishes were placed in a styrofoam box where water was evaporated at constant temperature, and the salt solution became oversaturated. A picture of the CuSO_4_ crystal growth on the Nafion plate is shown in Figure 1.

X-ray patterns of the crystal deposits were obtained with a Bruker D8 Discover A25 DaVinci Design diffractometer. The characteristics of this diffractometer are the following. The source of radiation was a Siemens KFL ceramic X-ray tube with a focus area of 0.4 × 12 mm^2^. The recording mode was as follows: CuKα radiation, Kβ-filter, *U* = 40 kV, *I* = 40 mA, Bragg–Brentano geometry, Soller collimators at 2.5°, a divergence slit of 0.638 mm, LYNXEYE detector, a scanning range 2θ = 10–65°, a scan step of 0.01°, and an exposure time is 7.5 s at each step. The spectra were processed with EVA software, version 2.1, and interpreted with the use of a PDF-2 database, 2011 version.

In addition, the rates of crystal growth on both smooth and polymer substrates were estimated. For this purpose, the *n*(*t*) refractive indices of the solutions were measured every 10 h with the use of an Abbe refractometer, Kruss AR4 (A.KRÜSS Optronic GmbH, Hamburg, Germany). For that purpose, a drop of the test solution with a volume of 5 μL was taken with an Acura 825 sampling microtube (Socorex Isba SA, Ecublens, Switzerland) and placed on the surface of the main measuring prism of the refractometer. The value of the refractive index was measured at the wavelength λ = 589.3 nm.

For the interpretation of experimental results, non-empirical quantum chemical simulations were used. Model fragments of a Nafion structure were calculated at the DFT level with the use of B3LYP hybrid exchange-correlation functional and 6-31G(d,p) extended double zeta basis set. To take account of the dispersion interactions that may be important within the perfluorinated segments of the model structures, D3+ Grimme correction was added. Such an approach was repeatedly shown to adequately describe the electron density distribution of both hydrocarbons or substituted hydrocarbons and water clusters due to the balanced account for the hydrogen bonding, electrostatic, and dispersion effects. The large atomic sizes of the model systems along with their spatial compactness make the usage of larger basis sets impossible (because of the rapidly decreasing parameter of the linear independence of the basis functions) and unnecessary (because the role of additional functions both with smaller exponents and larger angular momenta is played by the tails of numerous basis functions centered on a large number of more or less distant nuclei of the model system).

For the analysis of the conformation changes of Nafion in the presence of water, complex systems that involved up to 60 water molecules were considered. For estimating the hydration energies, the difference between the energy of the Nafion–water complex and the sum of the energies of the individual model Nafion fragment and the relaxed water cluster were found, the counterpoise corrections for the basis set superposition errors being taken into account in a conventional variant.

The possible formation of salt fragments on the surface of a Nafion membrane was mimicked by cluster systems, which involved a model Nafion structure, with up to five cation-anion pairs of the salt molecule initially arranged as in a crystal lattice, and the corresponding amount of water molecules. Here, restrictions on the positions of water molecules were imposed in order to prevent their agglomeration via H-bond formation, which was found to be probable and actual in the systems that comprise a relatively small number of particles insufficient for the formation of ordered arrangements typical of large ensembles and crystals. In the absence of salt or sucrose particles, there was no position or symmetry restriction during the optimization of the structures studied. In the latter case, the correspondence of the structures identified as a result of the optimization procedure to the local minima of the adiabatic potential was confirmed by normal-coordinate analysis.

To judge the relative stability of the systems, not only their total electronic energies (*E*) were estimated, but also thermal corrections were taken into account, and relative Δ*G_vib_* Gibbs energy values were found, which included the zero-point contributions and the vibrational energy increments (all the rotational and translational increments were neglected to mimic the actual highly restricted reciprocal mobility of the fragments of a polymer chain). All simulations were carried out with the use of the Firefly 8.2 quantum chemical program [10] and visualized with Chemcraft software, version 1.8 (build 654b) [11].

## 3. Experimental Results

### 3.1. Absorption Spectra of Nafion When Soaked in Natural Water and DDW

First of all, let us consider the absorption spectra of a dry Nafion specimen and a specimen swollen in water. Insofar as swelling is a response predetermined by hydrophobic/hydrophilic interactions and to a certain extent dependent on the flexibility of the hydrogen-bond network of water and the relative mobility of protons, the effects produced by different water samples may differ. We compared the behavior in natural deionized water and deuterium-depleted water. The absorption was analyzed in a broad range of wavelengths of 200 to 900 nm where Nafion is known to absorb [9]. Figure 1 shows the differential spectral functions ∆(*t*) of the Nafion specimens swollen in two different water samples, where:

∆(*t*) = *G*_1_ + *G*_2_ − *G*_3_(*t*). (3)

Here, *G*_1_ is the spectrum of a dry Nafion specimen with a thickness of 175 μm; *G*_2_ is the spectrum of the water sample Nafion specimen was soaked in; and *G*_3_(*t*) is the actual spectrum of a swollen Nafion specimen which was soaked for a time of *t*. Here, *t* is a parameter, the values of which were varied from 1 to 4 h. In fact, the *G*_1_ − *G*_3_(*t*) difference stands for the changes in the absorbance of Nafion upon its swelling in water. The absorbance decreases because of the formation of pores in the polymer matrix during soaking and filling of the pores with water, the absorption of which in the visible and near-UV ranges is negligible. Insofar as the water absorbance *G*_2_ is added to the above difference, Equation (3) reflects the changes in the absorbance of the polymer matrix itself during its swelling.

**Figure 2:**
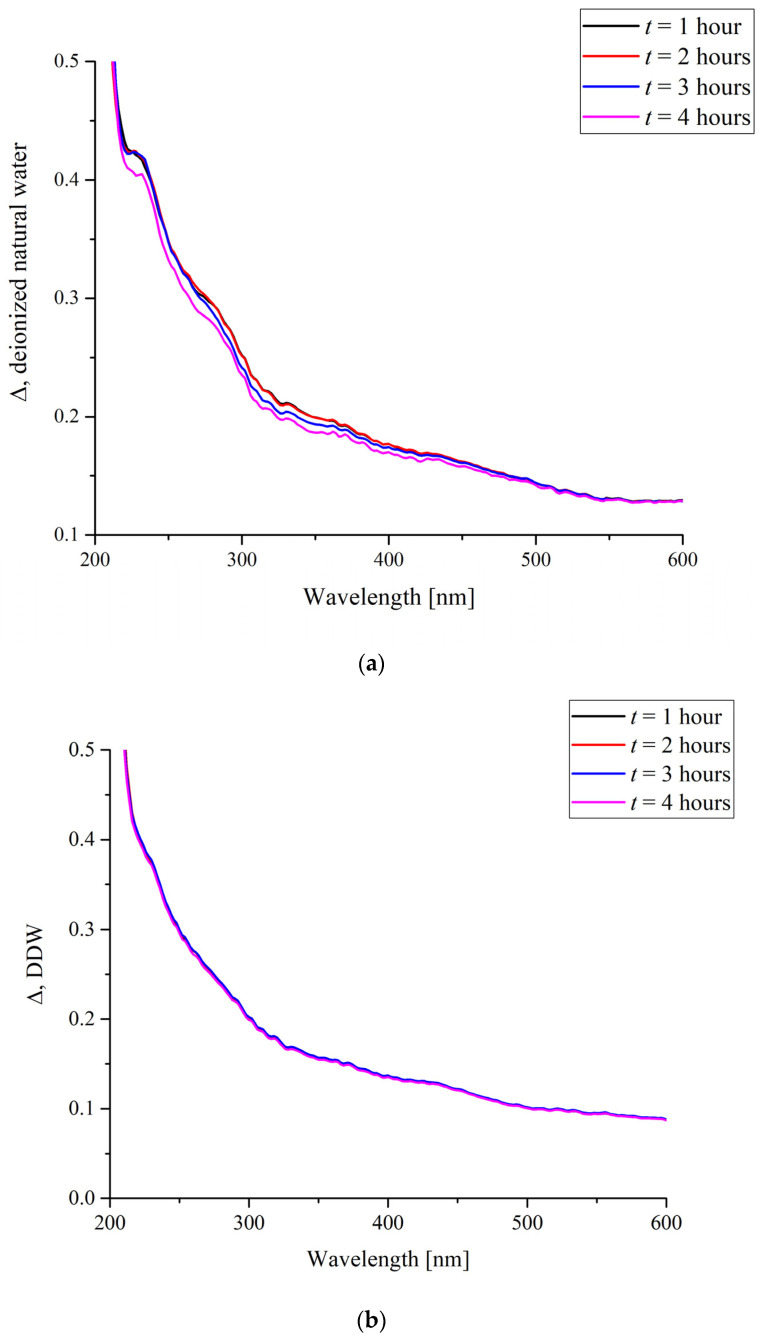
The differential absorbance of a Nafion membrane soaked in (**a**) natural deionized water and (**b**) deuterium-depleted water for *t* hours.

It was found that when a Nafion specimen was immersed in a DDW sample, its absorption remained nearly unchanged with time (Figure 2b), whereas contact with natural deionized water caused a gradual change in the spectrum (Figure 2a). The absorbance decreases with time within the wavelength range of 200 to 500 nm. Any change in the absorption reflects changes in the electronic structure of the specimen, which, at the same time, causes changes in the nuclear configuration. Thus, the decrease in absorbance in a relatively broad spectral range can be predetermined by the changes in the general conformation of chains when the neighborhoods of the groups of different chemical nature and, hence, their own structural parameters are changed. However, the absolute decrease in the absorbance is nearly the same within a subrange of 200 to 400 nm, which may mean that the absorption in this range is predetermined by the spectral response of the groups of the same kind with different internal configurations. Therefore, it is reasonable to consider an integral absorbance.

**Figure 3:**
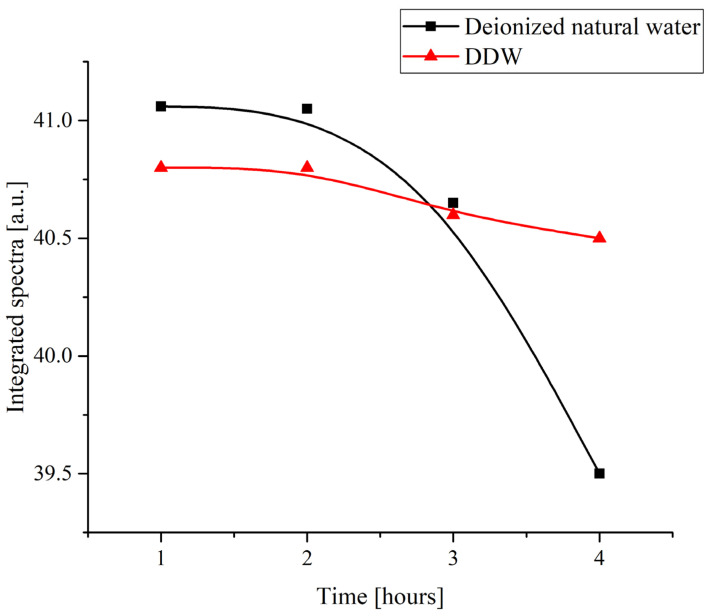
The integral absorbance of a Nafion membrane in a range of 200 to 600 nm upon its swelling in water depending on the duration of soaking.

Figure 3 shows the time dependence of the total absorbance of the specimens within the range of 200 to 600 nm obtained by numerical integration of the spectra shown in Figure 2. The duration of soaking was varied from 1 to 4 h.

This result even more clearly shows that natural deionized water produces a more pronounced effect on the Nafion polymer. The nature of the changes cannot be determined from such an experiment, but it can be said that the change in the deuterium content in water affects the character of the interaction between the water and the polymer. It is the surface state that, on one hand, is probably changed in water samples with a standard deuterium content and, at the same time, should affect any superficial processes such as deposition of substances. Then, the hypothesis about the changes in the surface state of the polymer and the character of the changes can be checked in an experiment in which different substances are crystallized on a Nafion surface being deposited from the corresponding supersaturated solutions.

This idea is further supported by the following rationale. If distances between Nafion fibers in water are about several nanometers (as suggested in [12]), and the edges of crystal lattice cells are no larger than a nanometer, the changes in the surface state of the polymer in water should affect the character of crystallization.

### 3.2. Experiments with X-ray Diffractometry of Crystalline Deposits on Nafion Substrate

**Figure 4:**
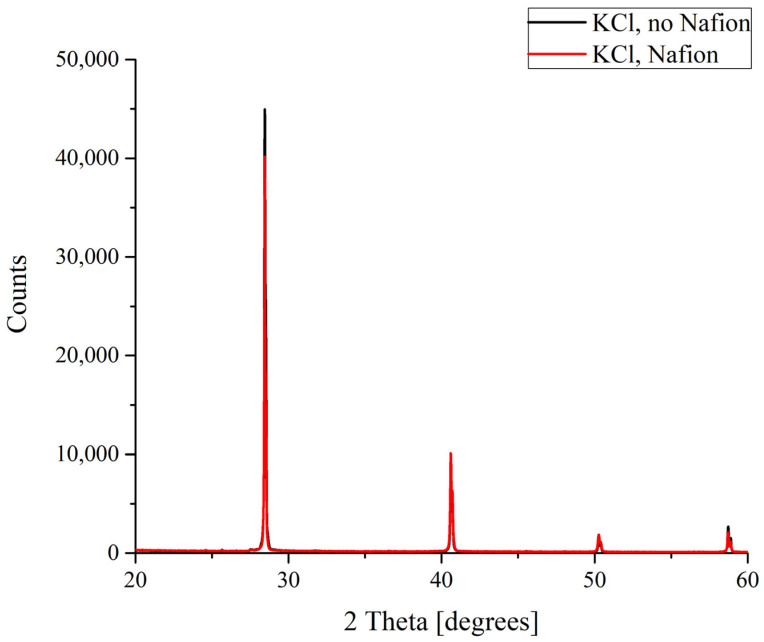
XRD patterns of KCl deposits formed in the presence and in the absence of Nafion plate.

**Figure 5:**
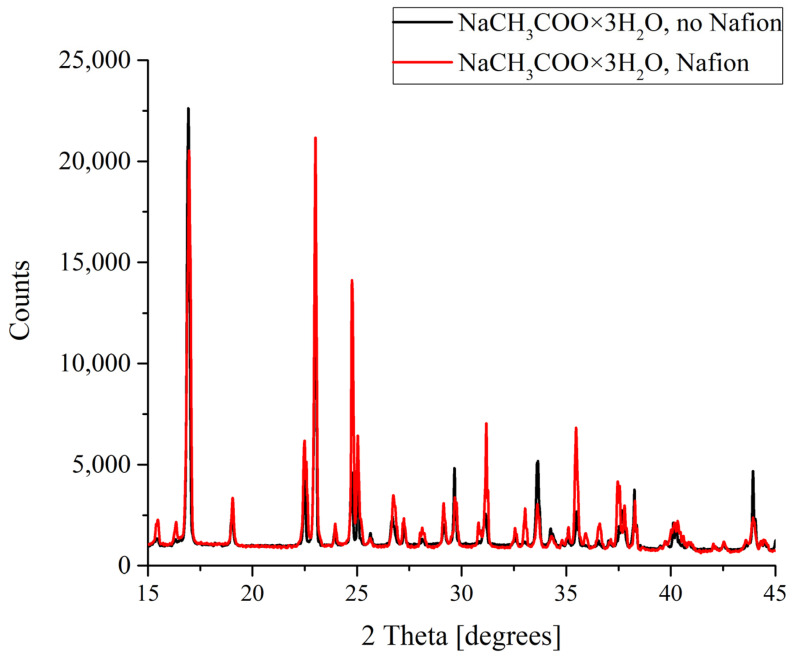
XRD patterns of NaCH_3_COO deposits formed in the presence and in the absence of Nafion plate.

**Figure 6:**
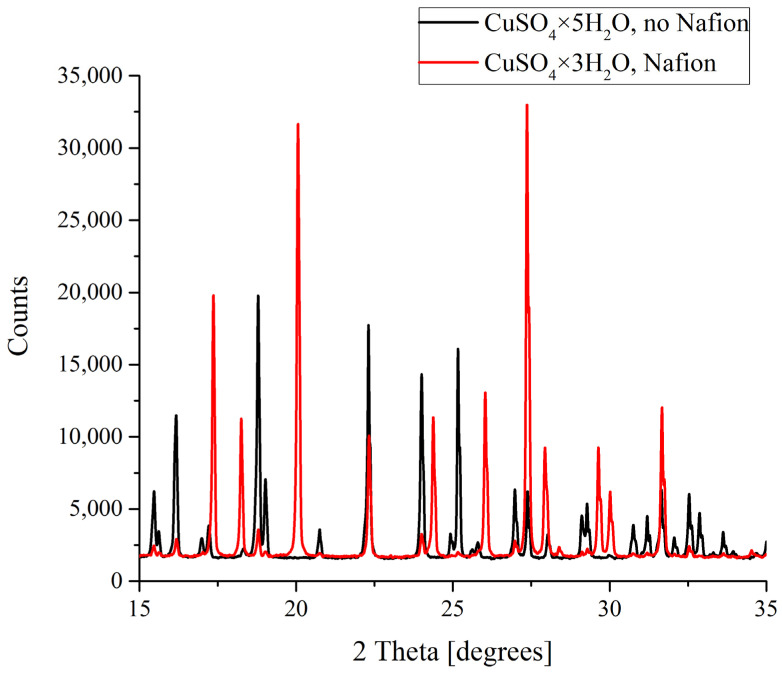
XRD patterns of CuSO_4_ deposits formed in the presence and in the absence of Nafion plate.

**Figure 7:**
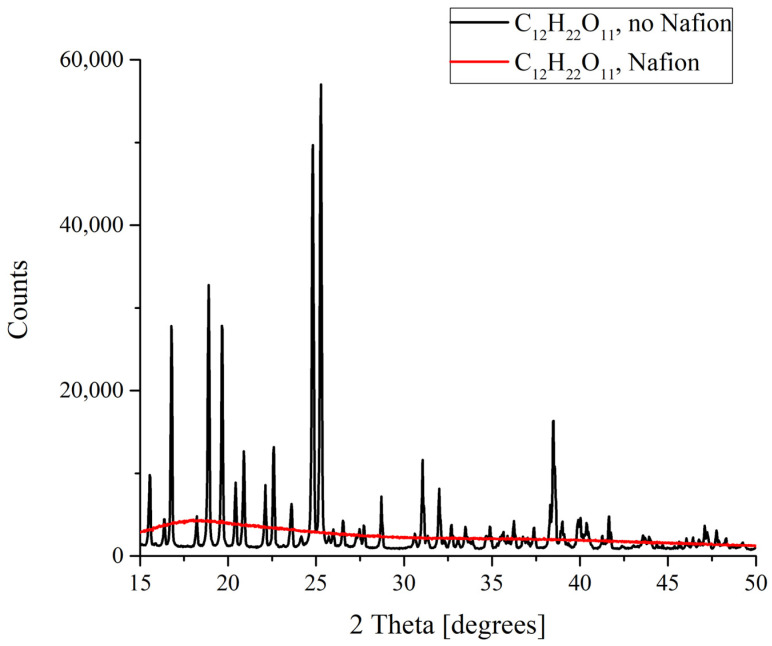
XRD patterns of sucrose C_12_H_22_O_11_ deposits formed in the presence and in the absence of Nafion plate.

The results of the X-ray diffractometry (XRD) of salt deposits formed in the solutions prepared with the use of natural deionized water either in the presence or in the absence of a Nafion plate in a Petri dish are shown in Figure 4, Figure 5, Figure 6 and Figure 7. Black curves correspond to the absence of a Nafion plate in a Petri dish, while red ones correspond to the presence of a plate in the dish. The abscissa-axis values are doubled X-ray diffraction angles, and the ordinate-axis values are the photocounts (the intensity of spectral signals). It is worth noting that there was no difference in the diffraction patterns obtained for the deposits formed on a smooth surface (in the absence of a Nafion plate) in the solutions based on natural deionized and deuterium-depleted water. Furthermore, there was no difference between the patterns of deposits formed in DDW on the smooth and polymer surfaces. Therefore, the results obtained in DDW-based solutions are not shown.

### 3.3. Dynamics of the Refractive Index of a Supersaturated Solution during the Formation of a Crystal on the Nafion Surface

**Figure 8:**
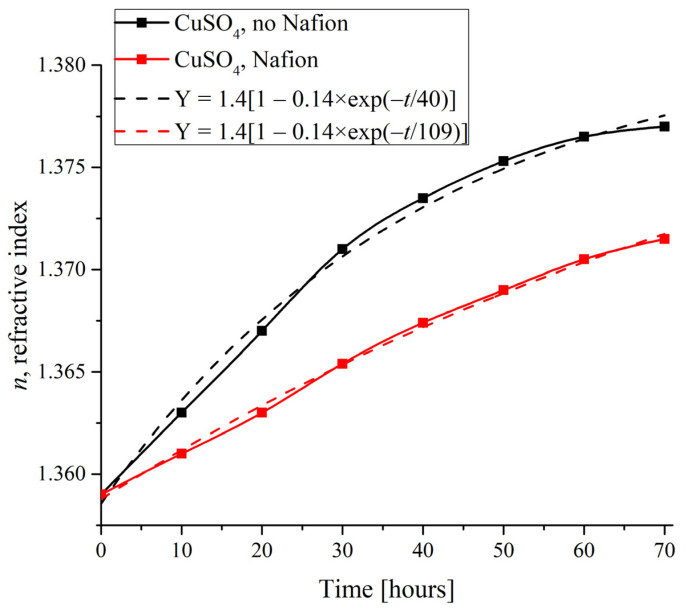
Refractive index of the CuSO_4_ specimen depending on the duration of deposit formation.

To compare the dynamics of crystalline deposition on the polymer substrate with the supposedly unwound polymer fibers and on a smooth glass surface, it is reasonable to study the changes in the physical characteristics of specimens with time in the presence and in the absence of a Nafion plate in the Petri dish. As an illustrative substance, copper sulfate was selected due to the formation of regular crystal lattices of definitely different symmetry on different substrates, and as a physical characteristic, an index of refraction was taken. Figure 8 shows the measured *n*(*t*) time dependences for the CuSO_4_ solutions based on natural deionized water when the deposits were formed on a Nafion plate and on the smooth surface of a dish.

## 4. Discussion

### 4.1. Discussion of Absorption Spectra of Nafion upon Swelling in Natural Water and DDW

As can be seen in Figure 3, the integral absorbance in natural deionized water drops after a certain characteristic duration of soaking *t_char_* = 2 h, whereas in a DDW sample, the integral absorbance remains nearly constant. Thus, the time of growing a rigid brush of unwound fibers on the surface of Nafion swelling in natural water is apparently about 2 h. Since the time of formation of a crystalline deposit from a supersaturated solution on a smooth glass substrate and on the surface of a polymer was several days, we can say that in the experiments on growing crystals from supersaturated solutions based on natural deionized water, unwinding of polymer fibers should manifest itself, which can affect the shape of the unit cell of the crystalline deposit on the polymer substrate.

### 4.2. Discussion of the Results of X-ray Diffractometry Experiments

As was shown in the previous section, we studied the growth of crystals with different unit cells of different systems from supersaturated solutions based on natural water and DDW. Let us recall that we have studied potassium chloride with a cubic crystal lattice (*a* = *b* = *c* = 3.63 Å; α = 90°, β = 90°, γ = 90°) [13]; sodium acetate trihydrate with a monoclinic lattice (*a* = 12.28–12.48 Å, *b* = 10.41–10.47 Å, *c* = 10.36–10.45 Å; α = 90°, β = 111.39–112.65°, γ = 90°) [14,15,16,17,18]; and copper sulfate that can crystallize as pentahydrate in a triclinic lattice (*a* = 5.97 Å, *b* = 6.10 Å, *c* = 10.64 Å; α = 77.2°, β = 82.4°, γ = 72.4°) and as trihydrate in a monoclinic lattice (*a* = 5.57 Å, *b* = 12.98 Å, *c* = 7.38 Å; α = 90°, β = 96.5°, γ = 90°) [19], as well as sucrose C_12_H_22_O_11_ with a monoclinic lattice (*a* = 7.72–7.77 Å, *b* = 8.68–8.71 Å, *c* = 10.82–10.88 Å, α = 90°, β = 102.9°, γ = 90°) [20,21].

In the case of potassium chloride, which crystallizes to form a cubic lattice solely that does not involve water molecules, no difference was found between the diffraction patterns of the crystals grown on smooth and polymer substrates (Figure 4).

For sodium acetate, again both diffraction patterns recorded for the deposits formed in the presence and in the absence of a Nafion plate in the Petri dish unambiguously reflected the appearance of a regular trihydrate (Figure 5). Note also that while the diffraction patterns overlap and nearly coincide in the case of KCl crystallization, in the case of NaCH_3_COO·3H_2_O we observe a broadening and a slight shift of the profile.

In the case of copper sulfate solutions based on the natural deionized water, a clear difference between the formation of crystals was observed. As was mentioned above, CuSO_4_ crystals grown in a supersaturated aqueous solution can be either pentahydrates (CuSO_4_·5H_2_O) with a triclinic lattice or trihydrates (CuSO_4_·3H_2_O) with a monoclinic lattice. As follows from the data shown in Figure 6, it is copper sulfate trihydrate that grows on a Nafion polymeric plate, while a smooth surface promotes the formation of the pentahydrate. Let us stress that in DDW-based solutions a crystalline deposit of copper sulfate pentahydrate with a triclinic lattice was formed irrespectively of whether a Nafion plate was placed in the Petri dish or not.

Finally, drastic differences in the formation of deposits from the saturated solutions were discovered for sucrose C_12_H_22_O_11_. It is well known that sucrose exists in the form of monoclinic crystals. When melted sucrose is cooled down, a transparent amorphous bulk named caramel is formed. Judging from the results obtained, it is caramel that forms as a result of the deposition on a Nafion plate (Figure 7). This means that in this case there is something that prevents an ordered deposition of particles., and this is probably the existence of more or less extended polymer fibers in the nucleation region which promote the strong disordering of crystal lattice cells and the resulting amorphizing.

Despite its relative compactness, the set of compounds we use can be considered as quite representative, since they have principally different syngonies, different molecular sizes, and different kinds of involvement of water molecules. Potassium chloride crystallizes with no lattice water, and eight (absolutely equivalent) K…Cl distances of about 3.15 Å, while Cl…Cl distances are proportional to 3.63 and 5.14 Å depending on the lattice direction. Sodium acetate can be anhydrous, but its typical stable form involves water molecules, each sodium atom having five neighboring molecules at a mean Na…O distance of 2.42 Å; but the local coordination is not highly symmetric, since the totally six oxygen neighbors of sodium (those of five water molecules and one acetate group) form a distorted octahedron with the Na…O distances varying in a range of 2.34 to 2.50 Å, which is quite typical of sodium [22]. Copper sulfate hydrates are very regular and at the same time involve water molecules. Additionally, the counterion in this case is complementary to the terminal SO_3_H groups of Nafion. As to the structural parameters, in trihydrate, the first shell of each copper atom involves three water molecules and three sulfate groups, whereas in pentahydrate, there are four water molecules and two sulfate groups, sulfates opposing each other in the extended octahedral-like configuration. Thus, the latter configuration can be treated as the one with the highest local symmetry among all three salt hydrates, while that of sodium acetate can be treated as the least structured. Finally, sucrose C_12_H_22_O_11_ is a relatively large molecule that involves penta- and hexamolecular cycles with numerous hydroxyl groups, which may cause certain spatial effects.

As shown below, all of the experimental results can be explained based on the nanoscale consideration of the systems. The most interesting result concerns copper sulfate. Because of the different syngony of its tri- and pentahydrates, the initial hypothesis was the following. The presence of a Nafion specimen in a Petri dish probably favors the formation of crystal lattices of a particular kind. Insofar as the key difference between the tri- and pentahydrate is the equality of one lattice angle to 90° in the former case, it was reasonable to assume that the orientation of the deposit growing on a Nafion surface is somehow spatially restricted in the tangential direction. This may be possible if there are some Nafion fibers that are oriented normally to the mean surface. This general peculiarity should be manifested in other situations as well. Two other salts considered in the work were potassium chloride and sodium acetate, which are crystallized (as noticed above) in cubic and monoclinic lattices. Both crystal lattices are complementary to the normal orientation of Nafion fibers with respect to the mean surface of the specimen, which means that the presence of Nafion may only either slightly distort or, by contrast, support the formation of those lattices depending on the complementarity of the parameters of the crystal lattice cells and the actual distances between the Nafion fibers. However, the crystal lattice of sucrose is of the same symmetry as that of the copper sulfate trihydrate. Then, the normal orientation of the fibers is not the sole condition that predetermines the crystal growth, and the arrangement of the fibers may be no less important.

**Figure 9:**
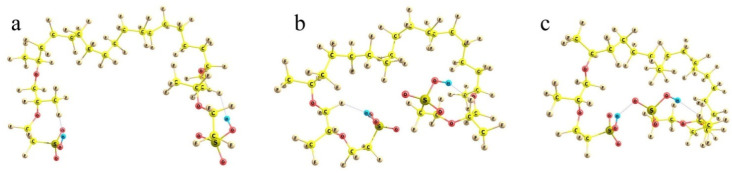
Three principally different conformations of the two-tail segment of the actual Nafion fiber. See text for details. Panel (**a**): the highest-energy structure with the most extended −SO_3_H-terminated side chains; Panel (**b**): lower-energy structure with the partly folded side chains; Panel (**c**): the lowest-energy structure with the side chains H-bonded via terminal −SO_3_H groups.

To check the idea, non-empirical simulations of the model Nafion fragments either individually or in combination with water molecules and cation-anion salt pairs were carried out. In accordance with the general Formula (1), in which *m* = 6.5 for Nafion N117 used in this work, a fragment selected for modeling involved the perfluorinated hydrocarbon skeleton with two side perfluorinated vinyl ether chains terminated by SO_3_H groups and two terminal methyl groups, so that the general composition of the fragment was CF_3_C(F)(O-CF_2_CF(CF_3_)OCF_2_CF_2_SO_3_H)(CF_2_)_14_C(F)(O-CF_2_CF(CF_3_)OCF_2_CF_2_SO_3_H)CF_3_ (Figure 9). At first, various conformations of the model were considered.

Because of the very large number of the degrees of freedom of the system considered related to the internal rotations around all single bonds, it is nearly impossible to find the absolute energy minimum of the system. However, judging from the known peculiarities of hydrocarbons, it is clear that mutual rotations of the neighboring functional groups, which involve atoms of the same kind (fluorine in our case), result in energetically either equivalent or close minima. The small differences appear due to the interactions with the more distant groups, all of which are of the prevailing dispersion nature. Such interactions provide contributions of no larger than a kcal/mole. At the same time, there are SO_3_H-ended chains, which should interact electrostatically; and these effects are much larger. Then, any set of the structures with the principally different arrangement of the side chains can give tentative estimates of the relative stability of those conformations. In any actual system, numerous interactions between the neighboring groups should inevitably distort any such structure; but the general energy trends should be the same. And the first result obtained is reasonable and quite expected. The more folded the branches and the closer the terminal sulfonate groups to each other, the lower the energy of the system. It decreases on going from (a) to (b) and (c) configurations in Figure 9.

When the SO_3_H-ended chains are initially kept at the largest possible distance from each other (taking into account that the structure should be extended in both directions, and any group has neighbors from both sides, the mean angles between the direction of the hydrophobic backbone and its polyether side chain should not exceed 90°) and extended to the utmost degree, the energy of the system is the highest (Figure 9a). When the branches are initially intorted, they tend to approach each other to minimize the net dipole moment (Figure 9b); and the electronic energy difference between these two structures is ca. 15 kcal/mol. Finally, if the terminal groups can form an H-bond, the stabilization of the system is maximum. The structure shown in Figure 9c is lower than the previous one by another 10 kcal/mol, which makes it already definitely favorable compared to the one with extended terminal branches (with an energy difference of ca. −25 kcal/mol). When thermal increments to the energy determined also by the different folding of the backbone (which becomes progressively less extended as the SO_3_H-ended chains become involved in the interaction with each other) are taken into account, the energy differences between the structures are decreased, but remain quite substantial: the Δ*G_vib_* relative Gibbs energies are 0 vs. −11 vs. −19 kcal/mol for the (a), (b), and (c) structures, respectively.

Thus, one can assume that in a dry state, Nafion fibers should be packed in such a way that their SO_3_H-ended chains are intorted as strongly as possible (in the presence of the neighboring segments), while the sulfonate groups themselves tend to approach each other to form hydrogen bonds, which provide closed segments within the hydrophobic matrix. Naturally, the number of chains (and sulfonate groups) involved in one H-bonded knot can be larger than two, but restricted because of the spatial limitations, and the additional groups can be either those from the adjacent fibers or of the neighboring segments of the same fiber. This makes the whole structure quite strongly entangled.

Now, let us turn to the hydration of a Nafion plate. To mimic the effect produced by water molecules, it is reasonable to restrict the consideration to a single-chain segment of the above Nafion model, i.e., to just one side chain connected to the perfluorinated backbone, and analyze what happens when the number of water molecules is gradually increased. Again, it is absolutely impossible and unnecessary to find all the local arrangements of water molecules around the model fragment. The most important conditions to be met are the reasonably large number of hydrogen bonds formed between water molecules and the smallest possible number of OH groups uninvolved in the bonds. The former condition corresponds to the largest possible contribution of the H-bond energies (each no less than 5 kcal/mol) to the total energy of the system, while the latter is the condition of the overall stability of the system against external perturbations.

**Figure 10:**
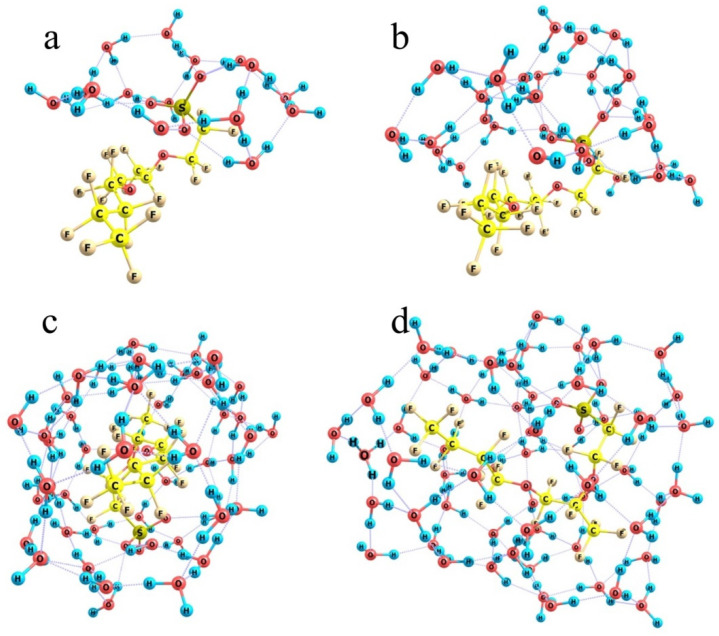
Optimized water shells around a single-chain Nafion fragment at the following n numbers of molecules. Panel (**a**): *n* = 16; Panel (**b**): *n* = 28; Panel (**c**): *n* = 50; Panel (**d**): *n* = 60.

When the number of molecules is small, they gather around the hydrophilic SO_3_H group (Figure 10a) and form a kind of a hat with an almost planar abat-jour visually parallel to the nearest contour of fluorine atoms. In the case of 16 molecules, which is sufficient for the formation of the first hydration shell around the SO_3_H group, the group dissociates (to form a hydronium ion and a negatively charged –SO_3_^−^ residue), and the corresponding energy decrease equals 49 kcal/mol, although the formal number of hydrogen bonds stabilizing the system is increased only by one, from 25 in the relaxed water cluster to 26 in the cluster bound to a hydrophilic head of the model Nafion fragment. Note that here the reference water cluster is the one that formed upon the relaxation of the aqueous coat upon removal of the model Nafion structure from it. If we would consider the lowest-energy water cluster composed of the same number of molecules, the energy gain upon the hydration of a model Nafion fragment is smaller, but insubstantially, by 5 kcal/mol, because the total number of hydrogen bonds that stabilize the cluster is larger only by one. The above figures mean that the H-bonds themselves are strengthened upon the reorganization of water molecules around the appeared proton and the negatively charged –SO_3_^−^ residue, and the mean solvation energy per one terminal group at a low water content can be estimated as ca. 45 kcal/mol.

When the number of water molecules is increased nearly two-fold (Figure 10b) and they are initially randomly arranged around the model Nafion fragment, the number of molecules in the close neighborhood of the –SO_3_^−^ residue is not substantially increased. The crown of its hat becomes thicker, and concurrently a water shell forms over the fluorocarbon chain separated from it by about 2.5 Å on the average. Water molecules are oriented in such a way that only some of the peripheral ones have dangling OH groups, while all the protons of the so-to-speak inner-structure molecules are involved in hydrogen bonds. This extension of the water shell corresponds to the formal total hydration energy of the model Nafion structure of 24.5 kcal/mol if the reference water cluster corresponds to a compact fragment of the bulk H-bond network. At the same time, if we would take the water cluster obtained upon the relaxation of the hydration shell of a model Nafion structure as the reference, the estimated energy gain would be much larger, about 50.4 kcal/mol. It means that when water molecules are rearranged around fluorocarbon chains, much energy is spent on the distortion of the original H-bond network of water. If it is compensated by the efficient hydration of hydronium ions and –SO_3_^−^ residues, the process is thermodynamically possible, but it gradually becomes less favorable as the length of fluorocarbon chains to be coated is increased. This trend becomes ultimately clear at the larger amount of water.

**Figure 11:**
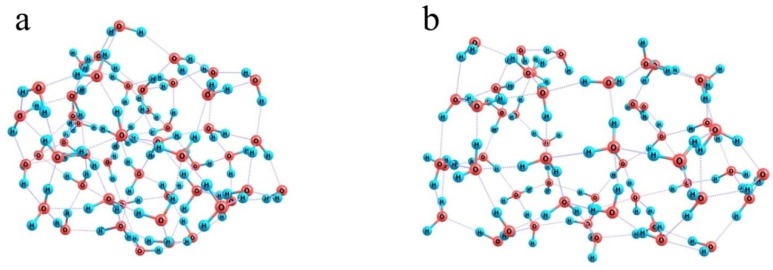
Illustration of the ultimate effect produced by the inclusion of a model Nafion fragment in an ensemble composed of 50 water molecules. Panel (**a**): a relaxed water cluster; Panel (**b**): a water shell of the model Nafion fragment.

When the number of water molecules is increased to half a hundred (Figure 10c) and they are initially randomly distributed around a model Nafion fragment, they can already form a closed monomolecular layer with the –SO_3_^−^ residue naturally built in this layer and the fluorocarbon chain residing in a kind of a tunnel; and a continuous network of 85 H-bonds is observed. However, this variant is not energetically favorable. Formation of such a water coat requires a dramatic distortion of the hydrogen-bond network of water. Instead of a compact ensemble of water molecules stabilized by a three-dimensional network of intermolecular bonds (Figure 11a), a strongly expanded two-dimensional bubble (Figure 11b) should appear, which is not additionally stabilized by the interactions with the structure it covers except for the coordination to an –SO_3_^−^ residue. Therefore, the energy difference between the hydrated Nafion fragment (Figure 10c) and a combination of the individual Nafion fragment and the relaxed water cluster (Figure 11a) equals 48 kcal/mol. Formally, this difference can be explained by the smaller number of hydrogen bonds the 2D bubble is stabilized with, namely 85 vs. 94 in the 3D ensemble, which accounts for about 5.3 kcal/mol per one additional bond. This is not an individual bond energy because of the collective effects typical of hydrogen-bond networks. Nevertheless, this is a reasonable estimate of the energy that should be supplied to the water system in one way or another to provide the necessary reorganization of hydrogen bonds. Note that it is nearly equal to the energy liberated upon the hydration of the –SO_3_^−^ residue, which means that the extension of hydration shells over the fluorocarbon chains is thermodynamically possible only at the cost of the hydration of hydrophilic segments, and the most energetically favorable variant is the one when only –SO_3_^−^ residues and their close neighbors are hydrated.

An additional argument in favor of such a conclusion is that even at the smallest considered number of water molecules, the SO_3_H group is dissociated (see above), and an H_3_O^+^ fragment separated from the –SO_3_^−^ residue with one water molecule appears. Insofar as all water molecules in the above systems are spent on the formation of a hydrating monolayer, there is no driving force for the migration of a detached proton to a larger distance from the –SO_3_^−^ residue. Only when the number of water molecules increases to 60, and some of the molecules act as nucleating sites of the second hydration shell, do there appear migration paths for the proton, and its departure from the –SO_3_^−^ residue becomes possible (Figure 10d); and in the optimum structure it resides close to the end of the fluorocarbon chain.

These results show that the hydration of side chains of a Nafion fiber is energetically favorable when water molecules do not penetrate deep and close to the perfluorinated Teflon backbone, and the energy necessary for the unfolding and extension of the fibers can easily be compensated by the hydration energy, especially taking into account that in this situation water molecules can be arranged at a larger distance from the perfluorinated carbon skeleton, which is also energetically favorable.

Thus, based on these model simulations, we can state that hydration of a Nafion plate should result in the unfolding of the side chains involving terminal SO_3_H groups, and the orientation of the chains should minimize the electrostatic repulsion of the residues and balance all the ionic interactions, which is best at their normal orientation to the Teflon backbone. Based on this conclusion, we can analyze the possible variants of the deposit crystal growth on such a surface.

**Figure 12:**
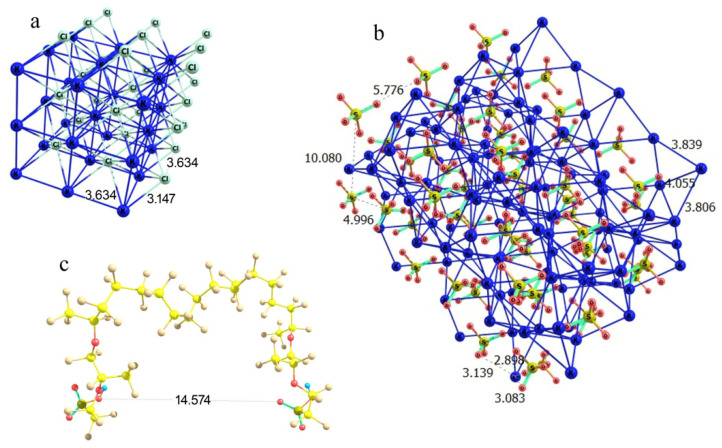
Schematic illustration of the partial complementarity of (**a**) KCl and (**b**) K_2_SO_4_ crystal lattices to the chain separation in the Nafion structure (**c**). Panel (**a**) KCl lattice fragment; Panel (**b**): K_2_SO_4_ lattice fragment; and Panel (**c**): a proper configuration of the model double-chain Nafion fragment.

In the case of potassium chloride, the regular cubic lattice implies the basic Cl…Cl and K…K distances equal to 3.63 Å at K…Cl contacts of 3.15 Å (Figure 12a). This means that the lattice segment that can be formed between two Nafion fibers should be characterized by a boundary distance proportional to 3.63 Å. It is worth noting that there are –SO_3_^−^ residues, which play the role of coordinating sites at the ends of side Nafion chains, where potassium cations can be bound. Thus, formally –SO_3_^−^ residues should substitute a chloride ion at some boundary lattice points. Potassium sulfate is known to crystallize in an orthorhombic lattice with the following cell parameters: *a* = 7.46 Å, *b* = 10.08 Å, *c* = 5.78 Å, α = 90°, β = 90°, γ = 90° [23]. The orientation of the crystal axes is the same as in a cubic lattice of KCl; but the internuclear distances differ, falling in a range of 3.80 to 4.06 Å in the case of K…K and of 5.00 to 10.08 Å in the case of S…S, while the K…O contacts are all 2.9 to 3.1 Å (Figure 12b). Thus, the K…Cl and K…O distances in the potassium chloride and sulfate crystals are sufficiently close, which formally means that the replacement of one K…Cl contact at the fiber boundary with a K…O–S(O_2_) should cause a minor perturbation rather than a substantial distortion of the crystal lattice. Furthermore, Cl…Cl distances in a potassium chloride lattice are proportional to 3.63 Å, while the distance between the oxygen atoms of the terminal –SO_3_^−^ residues of the two neighboring perfluorinated ether chains of Nafion (when the SO_3_H groups have symmetrically equivalent orientation) is about 14.6 Å (Figure 12c), which is almost exactly a four-fold Cl…Cl distance. This means that a fragment of KCl lattice can almost exactly fit in between the two neighboring fibers with minor perturbation compared to the individual lattice of the substance. Then, the growth of a cubic KCl crystal on a Nafion plate should be straightforward, and the side Nafion chains act just as coordinating and slightly armoring peripheral inclusions.

**Figure 13:**
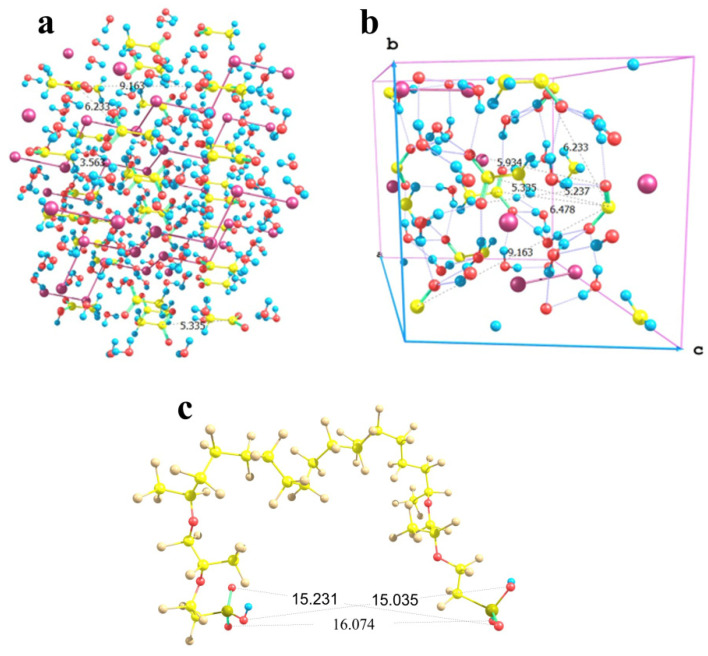
Schematic illustration of the minor correlation between the sodium acetate trihydrate crystal structure and the possible chain separation in the Nafion structure. Panel (**a**) CH_3_COONa × 3H_2_O lattice fragment; Panel (**b**): its basic cell with the b and c lattice vectors shown; and Panel (**c**): a proper configuration of the model double-chain Nafion fragment.

In the case of sodium acetate, the situation is more complex. Here, the counterion of the salt involves a CH_3_ group in addition to the carboxyl fragment. Those CH_3_ groups face each other in the crystal lattice being separated by a C…C distance of 3.56 Å (Figure 13a); and this is a strong limitation imposed on the flexibility of the particle arrangement. As a result, the orientations of the acetic groups are alternating in two orthogonal directions in the lattice. Taking into account that methyl groups cannot be located close to the Nafion fibers, the distances between the carbon atoms of those acetic groups, which have opposite orientations, are 6.23, 6.48, and 9.16 Å. No direction coincides with any lattice vector (Figure 13b), which means that the direction of crystal growth can by no means be driven by nearly parallel Nafion chains if they extend to the maximum possible degree and form a symmetric repeated brush on a Nafion surface. However, for the two acetic groups of the same orientation, the distance between the carbon atoms of their carboxyl groups is 5.34 Å, while the distances between the oxygen atoms of the groups are 5.24 and 5.93 Å. The latter values if increased threefold provide inter-nuclear distances of 15.72 and 17.79 Å as boundaries. In Nafion model conformations where both SO_3_H groups have the same spatial orientation, the O…O distances fall in a range of 15 to 16 Å (Figure 13c). Taking into account that the Nafion fibers are sufficiently flexible due to the possibility of numerous internal rotations within any perfluorinated chain, and their terminal groups can also change their positions especially under the effect of solvating water molecules, the distances between the terminal –SO_3_^−^ residues and the growing crystal can, so to speak, adapt to each other. However, if the orientations of the –SO_3_^−^ residues of the neighboring chains do not comply with the above requirement, the reorganization may require additional time and energy. As a result, the growing bottom part of the crystal (at least two cells in thickness) may be defective. It is this defectiveness that probably predetermines the aforementioned broadening and small shifts of the signals in the XRD patterns of the sodium acetate deposit grown on a Nafion substrate compared to that formed on a glass surface. Thus, in this situation, the fibers can by no means act as armoring elements but rather as superficial inclusions, which do not produce a noticeable distortion.

**Figure 14:**
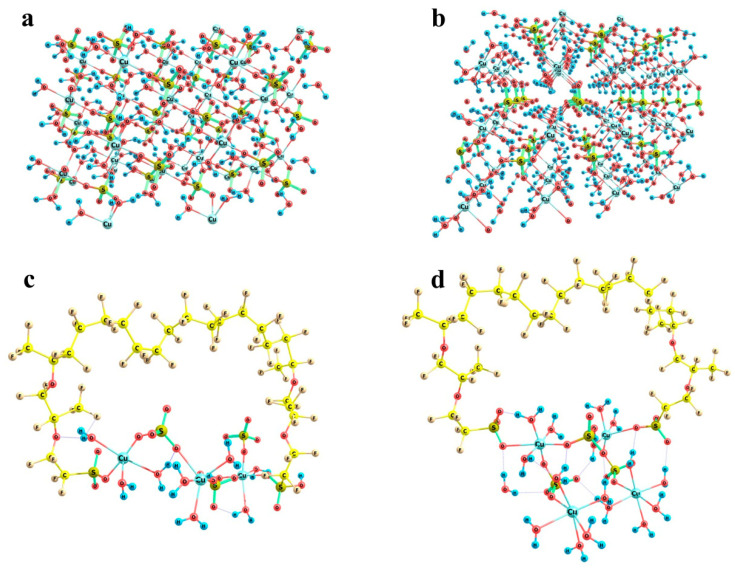
Fragments of the crystal structures of copper sulfate hydrates and the possibility of their building-in between Nafion side chains: Panel (**a**) CuSO_4_ × 3H_2_O lattice segment; Panel (**b**) CuSO_4_ × 5H_2_O lattice segment; Panel (**c**) (Cu)_3_(SO_4_)_3_(H_2_O)_9_ model cluster; Panel (**d**) (Cu)_4_(SO_4_)_3_(H_2_O)_16_ model cluster.

Now, let us turn to the most interesting example of copper sulfate salt. As was mentioned above, it can crystallize to form two different hydrates, namely trihydrate with a monoclinic lattice and pentahydrate with a triclinic lattice. Fragments of both crystal structures are shown in Figure 14a,b respectively. In trihydrate, the first shell of any copper atom involves three water molecules and three sulfate groups, so that the Cu…O distances are not equal (falling in ranges of 1.96 to 1.98 Å in the case of water oxygens and 1.96 to 2.45 Å in the case of sulfate oxygens), but the directions of bonds are quite regular in view of the replicated cells. In pentahydrate, the situation is quite similar, namely, the first shell of copper involves four water molecules and two sulfate groups, and the sulfates are arranged along a nearly C_4_ local symmetry axis (if only oxygens rather than whole water molecules are taken into account). As a result, here the local O-symmetry of a copper site corresponds to an elongated octahedron: two Cu…O distances to sulfates are equal to 2.41 Å, and Cu…O distances to water oxygens are equal in opposing pairs with a slight difference between the pairs (1.97 and 1.94 Å). The higher local symmetry of pentahydrate imposes stronger restrictions on the possible boundaries that may be imposed by Nafion chains, whereas the generally less symmetric trihydrate structure with the variations in the sulfate group orientations makes the problem of fitting of a crystal structure fragment to the space between Nafion chains more easily solvable.

Figure 14c,d illustrates the almost exact inclusion of such fragments. Two variants were considered, namely a (Cu)_3_(SO_4_)_3_(H_2_O)_9_ cluster (Figure 14c) and a (Cu)_4_(SO_4_)_3_(H_2_O)_16_ cluster (Figure 14d). The initial mutual arrangement of copper ions, sulfate groups, and water molecules in both combined model systems corresponded to the copper sulfate pentahydrate lattice. Then, the structures were optimized at the imposed restrictions on the mutual positions of copper and sulfur atoms of the sulfate groups, as well as the distances between copper atoms and water oxygens in order to prevent the agglomeration of water molecules and the approach of the distant oppositely charged ions, both of which are pronounced at such small relative numbers of water molecules in small ensembles of particles. The two systems of different size were considered in order to illustrate that rotations of the copper sulfate hydrate ensemble with respect to the Nafion chains can provide a compensation for the changes in the distances between the Nafion –SO_3_^−^ residues. The corresponding S…S distances in the combined model systems were 12.7 and 11.5 Å, respectively. This means that here Nafion chains can probably be built in the crystal deposit in the most efficient way.

**Figure 15:**
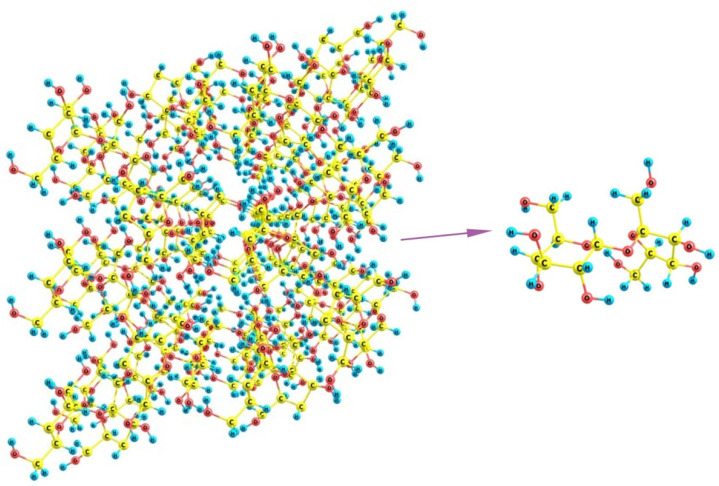
A fragment of the crystal lattice of sucrose C_12_H_22_O_11_ and the structure of its individual molecule.

Finally, if we turn to sucrose, it can be noticed that its crystals involve staircase brushes of alternating methylene groups and hydroxyls (Figure 15) where no distinct regions of predominantly hydrophilic or hydrophobic nature can be distinguished. Additionally, because of the large size of the molecules, which are nevertheless smaller than the above distances between the unfolded SO_3_-terminated chains of Nafion, there is no possibility for a regular fitting of sucrose molecule between the fibers. The sole variant is the wavy changes when some two neighboring chains are at a larger distance (about 16 Å between their terminal groups) and, thus, can accommodate two sucrose molecules in between, whereas the distances to their next left- and right-hand-side neighbors are much smaller, so that only one molecule can be located there. This variant is possible only when the orientation of Nafion fibers is not normal to the mean polymer surface, and these are inclined at varying angles. However, such dangling fibers are no longer a regular brush, which could stabilize some cubic, orthorhombic, or monoclinic lattice, but rather an undressed hair that could only distort any kind of molecular organization. Thus, the discrepancy between both individual sucrose molecules and their crystal packing, on one hand, and the possible regular arrangements of Nafion chains, on the other, should make the nucleation of the sucrose deposit strongly unordered and disturbed, which favors the appearance of an amorphous material instead of a crystal.

Summing up, Nafion differs from most coiled or folded polymers susceptible to swelling. Typically, an increase in the volume of the polymer matrix as a result of swelling causes certain mechanochemical or physical changes determined by the acquired inner strains. Usually, such behavior is demonstrated by cross-linked polymers, whose surfaces are not substantially affected by soaking. Nafion is not a cross-linked polymer, but rather a strongly branched one with a drastic difference with respect to hydration between the backbone and side chains. As shown at a nanometer level, these chains should be unfolded upon immersing the polymer in an aqueous solution. Their resulting extension is not as large, about 9 Å, of which only 6 Å segments are reasonably hydrophilic, and the behavior of the segments in water apparently plays a substantial role in the character of the surface processes.

Terminal SO_3_H groups are shown to be dissociated even in the presence of a small number of water molecules, which means that in actual solutions, the dissociation should always take place. The separation between the side chains, which is about 12 to 14 Å depending on the conformation of the backbone, makes the dissociation of nearly all the groups which are brought in contact with water highly probable. The dissociation in turn promotes further unfolding of other chains and their as regular as possible arrangement over the surface due to the governing balance of electrostatic forces. As a result, –SO_3_^−^ residues bonded to but separated from the hydrophobic backbone can act as a regular coordinating brush or grid. Their negative charges make them efficient coordinating sites for cations, and when the compound dissolved can crystallize in a lattice, an integer number of whose unit cells can fit in between the extended Nafion chains, crystallization is promoted, and Nafion acts as a true template.

By contrast to typical nearly planar templates, the Nafion template is characterized by a certain variability in the armoring parameters due to the flexibility of the chains. As a result, compounds with different unit cell parameters can be deposited on the Nafion surface. The sole, seemingly principal, restriction is that the syngony should be no lower than monoclinic because of the optimum normal orientation of the chains with respect to the backbone. This is illustrated by the examples of the formation of cubic potassium chloride and monoclinic sodium acetate trihydrate crystal deposits. In the case when the compound can be crystallized in triclinic and monoclinic syngonies, like copper sulfate hydrates, the above feature of Nafion makes the crystallization of monoclinic deposits preferable and selective. At the same time, if the unit cell parameters prevent a stable building of an integer number of the cells in between the Nafion chains and the chemical composition of the molecules is by no means complementary to the hydrophilic segments of the polymer fibers, the deposit becomes amorphous.

### 4.3. Discussion of the Results of the Refractometry Experiment

Insofar as the refractive index correlates with the salt concentration, its changes indirectly characterize the growth rate of the deposit. The dependences were found to be approximated with the following exponential function (see the inset in Figure 8):

Y = 1.4[1 − 0.14 × exp(−*t*/τ)]. (4)

Here we would like to refer to work [24], where an extensive study of the concentration dependence of the refractive index of aqueous CuSO_4_ solutions was carried out. Following the study [24], theoretical and experimental estimates of the refractive index of CuSO_4_ pentahydrate give *n* = 1.514, and the maximum value of the refractive index in supersaturated aqueous solutions of CuSO_4_ in [24] was *n* = 1.35 (see Table 1 in [24], where the results of measurements and theoretical calculations are presented), which is very close to the asymptotic value of our approximating curve: *n*(*t*) = 1.4 at *t* → ∞ (Equation (4)).

As shown in Figure 8, in the absence of a Nafion plate in a Petri dish, the characteristic time was estimated as τ = 40 h, while in the presence of a Nafion plate it increased to τ = 109 h, i.e., the refractive index on the surface of the solution more quickly reaches its equilibrium value *n* = 1.4 in the absence of the Nafion plate. Assuming that the evaporation of water from the surface of the solution occurs at the same rate under identical physical conditions, we can claim that the time τ indicates how rapidly the concentration of copper sulfate increases within the probe superficial region for compensating the depletion caused by the deposition. Since such a compensation is much faster in the absence of a Nafion plate in the Petri dish, the ratio of characteristic times in the presence to those in the absence of a Nafion plate can be taken as a rough estimate of the acceleration of the deposition process on the Nafion substrate, which is explained within the framework of the theoretical model, see Section 4.2. Besides that, in accordance with our observations, all studied crystals grew approximately twice as faster on the surface of Nafion compared to the case of absence of a Nafion plate in the Petri dish.

## 5. Conclusions

In X-ray diffraction experiments, the crystalline patterns grown on a Nafion substrate from supersaturated aqueous solutions based on natural water and DDW were studied. In accordance with the model we are developing here, these crystals were grown under the conditions of unwinding the polymer fibers into the bulk liquid, or in the absence of this effect. It turned out that the flexible brush of the fibers, formed at the Nafion interface when soaking in natural water, presets the geometry of the crystal unit cell, which was especially pronounced in the case of CuSO_4_. This salt has two crystal modifications, namely, pentahydrate and trihydrate, the pentahydrate being energetically more favorable, see Section 4.2. However, the unit cell of the pentahydrate has the shape of a skewed parallelepiped, while the unit cell of the trihydrate is a rectangular parallelepiped. Since the unwound fibers are oriented normally to the mean polymer surface, this predetermines the direction of one of the edges of the unit cell. As a result, predominantly CuSO_4_ trihydrate crystals grow on such a surface. Another drastic change produced by the Nafion substrate is the possible amorphization of the deposit, as was shown in the experiments with sucrose.

Thus, Nafion can act as an efficient template, which can either promote the formation of regular deposits, or provide conditions for the selective crystallization of a particular crystal form, or even act as an amorphizing agent.

## Data Availability

Data are contained within the article.
